# Investigation of METTL3 as an inhibitor of kanamycin-induced ototoxicity via stress granule formation

**DOI:** 10.3389/fphar.2024.1430162

**Published:** 2024-08-13

**Authors:** Yan Wu, Yu-Yu Huang, Lu-Yao Wang, Yan Yang, Fei-Lun Cui, Shu-Na Li

**Affiliations:** ^1^ Department of Otorhinolaryngology-Head and Neck Surgery, Xinhua Hospital, Shanghai Jiaotong University School of Medicine, Shanghai, China; ^2^ Shanghai Jiaotong University School of Medicine Ear Institute, Shanghai, China; ^3^ Shanghai Key Laboratory of Translational Medicine on Ear and Nose Diseases, Shanghai, China; ^4^ Liaoning Medical Device Test Institute, Shenyang, China; ^5^ Urology Department, Taizhou Second People’s Hospital Affiliated to Yangzhou University, Taizhou, China

**Keywords:** kanamycin, METTL3, ototoxicity, reactive oxygen species, stress granule

## Abstract

**Background:**

Methyltransferase-like 3 (METTL3), a component of the N6-methyladenosine (m6A) methyltransferase family, exhibits significant expression in HEI-OC1 cells and cochlear explants. Aminoglycoside antibiotics, known for their ototoxic potential, frequently induce irreversible auditory damage in hair cells, predominantly through oxidative stress mechanisms. However, the specific role of METTL3 in kanamycin-induced hair cell loss remains unclear.

**Objective:**

This study aims to elucidate the mechanisms by which METTL3 contributes to kanamycin-induced ototoxicity.

**Methods and Results:**

*In vivo* experiments demonstrated a notable reduction in METTL3 expression within cochlear explants following kanamycin administration, concomitant with the formation of stress granules (SGs). Similarly, a 24-hour kanamycin treatment led to decreased METTL3 expression and induced SG formation both in HEI-OC1 cells and neonatal cochlear explants, corroborating the *in vivo* observations. Lentivirus-mediated transfection was employed to overexpress and knockdown METTL3 in HEI-OC1 cells. Knockdown of METTL3 resulted in increased reactive oxygen species (ROS) levels and apoptosis induced by kanamycin, while concurrently reducing SG formation. Conversely, overexpression of METTL3 attenuated ROS generation, decreased apoptosis rates, and promoted SG formation induced by kanamycin. Therefore, METTL3-mediated SG formation presents a promising target for mitigating kanamycin-induced ROS generation and the rate of apoptosis.

**Conclusion:**

This finding indicates that METTL3-mediated SG formation holds potential in mitigating kanamycin-induced impairments in cochlear hair cells by reducing ROS formation and apoptosis rates.

## 1 Introduction

Globally, nearly one billion individuals suffer from deafness, a condition that imposes substantial burdens on both individuals and communities (GBD, 2019 Hearing Loss Collaborators, 2021). Hearing impairments can stem from various sources, including congenital, morphological, and developmental anomalies, aging, exposure to ototoxic drugs, intense noise, and genetic predispositions (Nieman and Oh, 2020). Despite the well-documented ototoxic effects of aminoglycoside antibiotics, such as kanamycin, their critical role in treating severe gram-negative bacterial infections and multidrug-resistant tuberculosis is indisputable (Poka-Mayap et al., 2020). The use of aminoglycosides is currently associated with the destruction of cochlear hair cells, presenting a significant challenge for both medical practitioners and patients (Gibaja et al., 2022; Li et al., 2023; Zhang et al., 2023). Significant advancements have been made in understanding the mechanisms involved in aminoglycoside antibiotic-induced ototoxicity, implicating factors such as reactive oxygen species (ROS), mitophagy, intrinsic programmed cell death, and epigenetic modifications (Kros and Steyger, 2019). Among these, oxidative stress is recognized as a pivotal mechanism (Tan and Song, 2023).

Epigenetics encompasses heritable changes in gene expression and function that do not involve alterations to the DNA base sequence (Jeffries, 2020). Recent research has focused on elucidating the mechanisms governing epigenetic modifications. Notably, over 100 RNA modifications have been identified, with N6-methyladenosine (m6A) being one of the most prevalent in eukaryotes (Huang et al., 2020). m6A methylation intricately regulates gene expression and has been demonstrated to be reversible, exhibiting dynamic changes across various developmental stages and tissues (Zong et al., 2019). The regulation of m6A modification is contingent upon the actions of m6A methyltransferases (writers), demethylases (erasers), and binding proteins (readers) (Chen WW. et al., 2020). Among these, METTL3, recognized as the principal methyltransferase, demonstrates high conservation across evolutionary scales, from yeast to humans, and plays a pivotal role in modulating RNA splicing, transportation, and translation governed by m6A (Rowe and Rockwell, 2022; Huang et al., 2023; Song et al., 2023).

**FIGURE 1:**
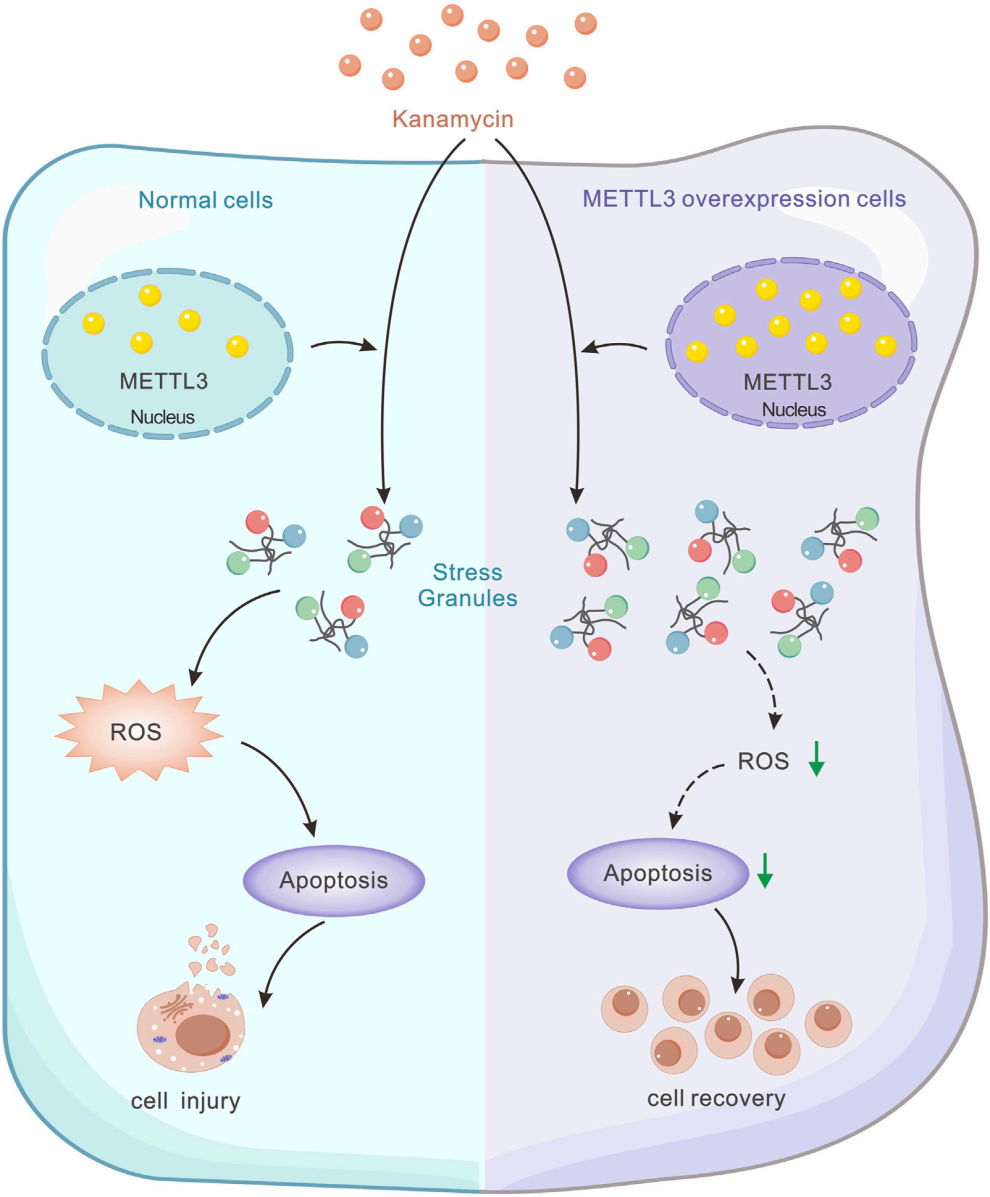
Schematic model illustrating the regulatory role of METTL3 in kanamycin-induced cell damage. Kanamycin treatment results in decreased METTL3 expression, while METTL3 functions to inhibit ROS-mediated apoptosis by promoting SG formation. This mechanism ultimately reduces kanamycin-induced cell ablation.

METTL3-mediated m6A methylation has been identified as a facilitator of miR-335 maturation, promoting stress granule (SG) assembly and mitigating apoptosis in acute ischemic stroke (Figure 1) (Si et al., 2020) Despite extensive studies on the interplay among METTL3, ROS, and apoptosis, its involvement in ototoxicity remains a subject of ongoing assessment (Wang et al., 2019; He et al., 2022).

Herein, we hypothesize that METTL3 may facilitate SG formation and alleviate oxidative stress induced by aminoglycosides in cochlear hair cells, thereby mitigating ototoxic injury. To assess this hypothesis, both *in vitro* and *in vivo* experiments were conducted. Comparative analyses were performed on cell viability, ROS levels, apoptotic signaling pathways, and SG formation in HEI-OC1 cells and cochlear hair cells subjected to kanamycin-induced ototoxicity, with and without METTL3 modulation.

## 2 Materials and methods

### 2.1 Animals

All mice (C57BL/6 background) were procured from the Laboratory Animal Center of Xinhua Hospital, affiliated with Shanghai Jiaotong University School of Medicine. The experiments involving mice were conducted following guidelines approved by the animal care institution of Shanghai Jiao Tong University. The mice were housed in a controlled environment with measures taken to minimize distress. Kanamycin powder (Sigma-Aldrich, St. Louis, MO, United States) was dissolved in 10 mL of sterile saline (0.9% NaCl) to achieve a concentration of 100 mg/mL. A single dose of kanamycin (1,000 mg/kg) followed by furosemide (100 mg/kg; Sigma-Aldrich, St. Louis, MO, United States) within 30 min, was administered to 6-week-old male C57BL/6 mice (Chen J. et al., 2020). The control group received sterile saline. All mice remained in satisfactory condition throughout the study.

### 2.2 Culture of the neonatal cochlear explants

Neonatal cochlear explants from P3 C57BL/6 mice were affixed to a small glass surface pretreated with Cell-Tak (Thermo Scientific, United States) and placed in an incubator at 37°C with 5% CO_2_. The explants were cultured in serum-free Dulbecco’s modified Eagle’s medium (DMEM) supplemented with 1% N-2 (Thermo Fisher Scientific, United States), 2% B-27 supplement (Invitrogen, United States), and 1% sodium ampicillin (Sangon, Shanghai, China). After 24 h, fresh medium containing 0.2 mM kanamycin was added, and the explants were further incubated for either 24 or 48 h. Following removal of the culture medium, the neonatal cochlear explants were fixed in 4% paraformaldehyde for subsequent experiments.

### 2.3 Cell cultures

The HEI-OC1 cell line, provided by Professor Chai Renjie (Southeast University), was maintained at 33°C in an atmosphere of 10% CO_2_. Cells were cultured in high-glucose DMEM (Gibco, United States) supplemented with 10% fetal bovine serum [FBS (Gibco, United States)]. For cell proliferation, the cells were detached using 0.25% trypsin/EDTA (Thermo Fisher Scientific, United States). Kanamycin (Sigma-Aldrich, United States) was dissolved in distilled water to achieve a concentration of 100 mM. After 24 h of culture without kanamycin, the culture medium was replaced with fresh medium containing 20 mM kanamycin and incubated for 24 h.

### 2.4 Cell viability

The cell viability of HEI-OC1 cells was assessed using a Cell Counting Kit-8 (CCK8; Beyotime, China) assay, following the instructions provided by the manufacturer. Briefly, cells were seeded in a 96-well plate at a density of 9,000 cells per well with five replicates and cultured overnight. The following day, the culture medium was replaced with fresh medium containing various concentrations of kanamycin (0, 1, 2, 5, 10, 15, and 20 mM), while the control group received DMEM without any treatment. After 24 h, the cells were incubated with CCK8 reagent for 2 h, and the optical density (OD) values were assessed at 450 nm. The cell survival rates under different concentrations of kanamycin were calculated as the ratio of OD values of the treated group to those of the control group.

### 2.5 Lentivirus transfection

All lentiviruses were sourced from Shanghai HanBio Co., Ltd. (Shanghai, China). The METTL3-gene cDNA, obtained through PCR cloning, was integrated into pHBLV-CMV-MCS-3flag-EF1-puromycin lentiviral vectors for METTL3 overexpression. Cells treated with the empty lentiviral vector were used as the control group. For gene knockdown, the sequences of METTL3 shRNA were GAT​CCG​CCT​CAG​TGG​ATC​TGT​TGT​GAT​CTC​GAG​ATC​ACA​ACA​GAT​CCA​CTG​AGG​TTT​TTT​G (shRNA-1); GAT​CCG​CGT​CAG​TAT​CTT​GGG​CAA​ATT​CTC​GAG​AAT​TTG​CCC​AAG​ATA​CTG​ACG​TTT​TTT​G (shRNA-2); and GAT​CCG​GCA​CCC​GCA​AGA​TTG​AGT​TAT​CTC​GAG​ATA​ACT​CAA​TCT​TGC​GGG​TGC​TTT​TTT​G (shRNA-3), which were also purchased from Shanghai HanBio Co., Ltd. (Shanghai, China). Cells transfected with negative shRNA (GAT​CCG​TTC​TCC​GAA​CGT​GTC​ACG​TAA​TTC​AAG​AGA​TTA​CGT​GAC​ACG​TTC​GGA​GAA​TTT​TTT​C) were used as a control.

HEI-OC1 cells were seeded into a 6-well plate at a density of 20,000 cells per well and exposed to a specific lentivirus when cell confluency reached 80%–90%. Following a 24-hour incubation period, the medium was replaced with fresh medium. Puromycin selection was initiated after 72 h of transduction using puromycin (Meilunbio, MA0318) until positive cell populations were obtained. Subsequently, METTL3-gene-overexpressing and knockdown cell lines were harvested for assessment of METTL3-gene expression levels through Western blotting assay.

### 2.6 Flow cytometry

Following kanamycin administration, apoptotic cells and ROS production were assessed using an Annexin/V-FITC apoptosis kit (Keygen, China) and ROS Assay kit (Beyotime, China), respectively, in accordance with the instructions provided by the manufacturer. The fluorescence intensity of each group was quantified using flow cytometry (FACS Calibur, BD Biosciences), and data analysis was conducted using FlowJo 7.6 software. Cells not subjected to ototoxic treatment were used as controls.

### 2.7 ROS experiment

The culture medium in the plates was substituted with fresh medium containing diluted DCFH‐DA. Following a 20-minute incubation at 37°C, excess DCFH‐DA was removed by rinsing, and the level of ROS accumulation was assessed using fluorescence. Quantification of DCFH‐DA in HEI-OC1 cells was performed by enumerating green fluorescent spots.

### 2.8 Immunofluorescence

Cochleae, obtained from the temporal bones of 6-week-old mice, were fixed in 4% paraformaldehyde (Beyotime, P0099) overnight at 37°C for whole mount and frozen section. Neonatal cochlear explants derived from postnatal day 3 mice were fixed in 4% paraformaldehyde at 37°C for 30 min. Following fixation and rinsing, the specimens were permeabilized using a 0.1% Triton X-100 solution for 30 min and blocked with 10% goat serum for 40 min. Primary antibodies (rabbit anti-METTL3 antibody, 1:1000, Abcam; mouse anti-myosin 7a antibody, 1:200, Santa Cruz; rabbit anti-TIA1 antibody, 1:1000, Abcam; mouse anti-G3BP1 antibody, 1:1000, Abcam) were then applied to the specimens for overnight incubation at 4°C, followed by secondary antibodies [Alexa Fluor 488 AffiniPure Goat Anti-Rabbit IgG (H + L), 1:200, YEASEN, China; Alexa Fluor 594 AffiniPure Goat Anti- Mouse IgG (H + L), 1:200, YEASEN, China] at 37°C for 2 h. Cochlear explants obtained from adult mice were treated with Phalloidin (YEASEN, China) for 20 min. Subsequently, DAPI (Beyotime, China) counterstaining was performed for 10 min. Samples were observed under a laser confocal microscope (Leica SP8, Germany) after being sealed with anti-fade fluorescence mounting medium, VECTASHIELD (Vector Laboratories, Canada). SGs in hair cells were quantified by enumerating fluorescent spots of TIA1, while SGs in HEI-OC1 cells were quantified by counting punctate fluorescent spots of TIA1 and G3BP1.

### 2.9 Western blotting analysis

Cochleae from mice aged 1–6 weeks were collected, with four cochleae per sample. For neonatal cochlear explants, four explants were harvested for each sample. The tissues were rinsed with PBS and then lysed in an ice-cold mixture comprising RIPA buffer, protease inhibitor cocktails, and PMSF on ice for 15 min at 4°C. Following harvesting, the samples were centrifuged at 12,000 rpm for 8 min, and the supernatant was retained to assess protein concentrations using the BCA kit (Beyotime, China). Total proteins were pretreated with 5X loading buffer and denatured at 95°C for 8 min. After separation on a 12% SDS-PAGE gel, protein samples (20 μg/well) were transferred onto a PVDF membrane (Thermo Fisher, United States). Subsequently, the membrane was soaked in a blocking buffer (Beyotime, China) and then incubated with primary antibodies (rabbit anti-METTL3 antibody, Abcam; rabbit anti-TIA1 antibody, Abcam; mouse anti-G3BP1 antibody, Abcam) overnight at 4°C. Following rinsing, the membrane was incubated with secondary antibodies conjugated with HRP for 1.5 h at room temperature. β-actin (Beyotime, China) was used as the internal control. Protein bands were visualized using ECL chemiluminescence kits (Beyotime, China), and images were captured on an Image Lab System (Bio‐Rad, United States). ImageJ software was used to open the TIFF image file of a band, convert the image into a grayscale image (16 bit), and eliminate its background impact (the parameter was set to 50). Measurements including area, average density, grayscale value, and integrated density were set, with the unit of length set to pixels. The image was then converted into a bright band, and the grayscale value of the target protein when compared to β-actin was calculated for semi-quantification.

### 2.10 Auditory brainstem response (ABR)

The mice (n = 4 for each group) were administered ketamine (150 mg/kg) and xylazine (6 mg/kg) intraperitoneally to induce general anesthesia. Following the attainment of adequate anesthesia, silver needle electrodes were subcutaneously positioned at the vertex (reference electrode), mastoid process (stimulating electrode), and contralateral thigh (ground electrode) within a soundproof chamber. The ABR thresholds were determined as the lowest stimulation intensity required to elicit replicable wave I. Heating pads were used to maintain the body temperature of the mice during anesthesia.

ABR tests were conducted using a Tucker Davis Technologies (TDT) RZ6 device and BiosigRZ software. ABR results were recorded in response to short pure tones of various frequencies (4, 8, 11, 16, 22, and 32 kHz). The maximum intensity of acoustic stimulation was set at 90 dB SPL.

### 2.11 Statistical analysis

All data are expressed as the mean ± standard error of the mean (SEM), and the experiments were conducted in triplicate. GraphPad Prism software was used for data analysis. Differences between more than two groups were analyzed using one-way analysis of variance (ANOVA). The unpaired, two-tailed t-test was used to compare differences between two independent groups.

## 3 Results

### 3.1 Kanamycin administration *in vivo* induced SG formation and downregulated METTL3 expression in the cochlea

**FIGURE 2:**
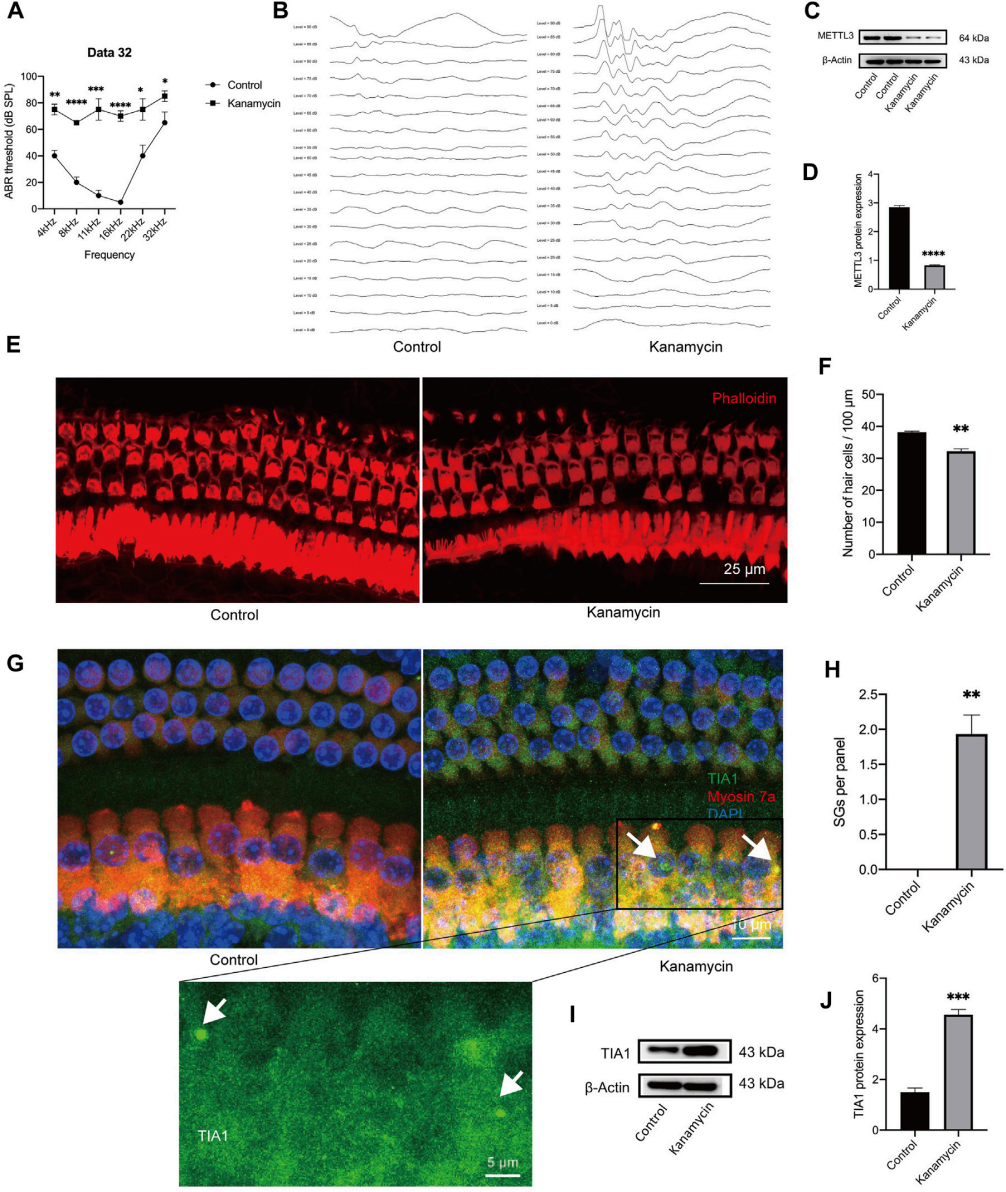
*In vivo* administration of aminoglycosides induces SG formation and decreases the expression level of METTL3 in the cochlea. The control group consisted of animals treated with sterile saline, while the kanamycin group comprised of animals treated with kanamycin/furosemide injections for 3 days. **(A, B)** Auditory Brainstem Response (ABR) thresholds at frequencies of 4, 8, 11, 16, 22, and 32 kHz were compared between the two groups. Representative images of ABR at 8 kHz for both groups are presented. **(C, D)** Significantly reduced METTL3 expression was observed in the kanamycin-treated group, as evidenced by Western blot analysis, which quantitatively demonstrated the differences in gray values between the various groups. **(E, F)** Phalloidin staining was used to mark isolated cochlear hair cells (red), followed by cell counting. **(G)** Immunostaining results displayed cochlear hair cell loss and the presence of SGs (indicated by white arrows) through the immunostaining of TIA1 (green), myosin 7a (red), and DAPI (blue) after kanamycin treatment. **(H)** Quantification analysis revealed SGs in cochlear explants from adult mice, represented as per 100 μm length of the cochlea. **(I, J)** Western blot analysis confirmed an increase in the expression of the SG marker, TIA1, in cochlear explants following kanamycin treatment. The quantitation method of Western blotting demonstrated the magnitude of gray values between different groups. Each group consisted of n = 4 samples; *P < 0.05; **P < 0.01; ***P < 0.001; ****P < 0.0001; vs. the control group. The unpaired, two-tailed t-test was used for statistical analysis.

Cochleae were collected on day 3 following systemic administration of kanamycin/furosemide or sterile saline. Hearing thresholds were elevated following kanamycin treatment (Figures 2A, B). Furthermore, kanamycin treatment significantly reduced METTL3 expression levels (Figures 2C, D). Examination of cochlear explants stained with phalloidin (red) revealed hair cell loss in the apical turn (Figures 2E, F). SGs are commonly identified using counterstaining with T-cell intracellular antigen-1 (TIA1) and GTPase-activating protein-binding protein 1 (G3BP1) in cell lines. As per Towers et al. and Gonçalves et al., SGs in hair cells can be labeled solely with TIA1 (Gonçalves et al., 2019b; Gonçalves et al., 2019a). Due to limitations of our laser confocal microscope, which could display only three fluorescence channels, staining was performed for DAPI, myosin 7a, and TIA1 without G3BP1. Elevated expression of TIA1 in hair cells following kanamycin treatment was confirmed (Figures 2G, H). Western blotting results indicated an increase in the expression level of TIA1 after kanamycin treatment (Figures 2I, J). These findings collectively suggest that kanamycin administration promotes SG expression and contributes to hair cell loss in mice.

### 3.2 METTL3 expression in neonatal cochlear explants and HEI-OC1 cells

**FIGURE 3:**
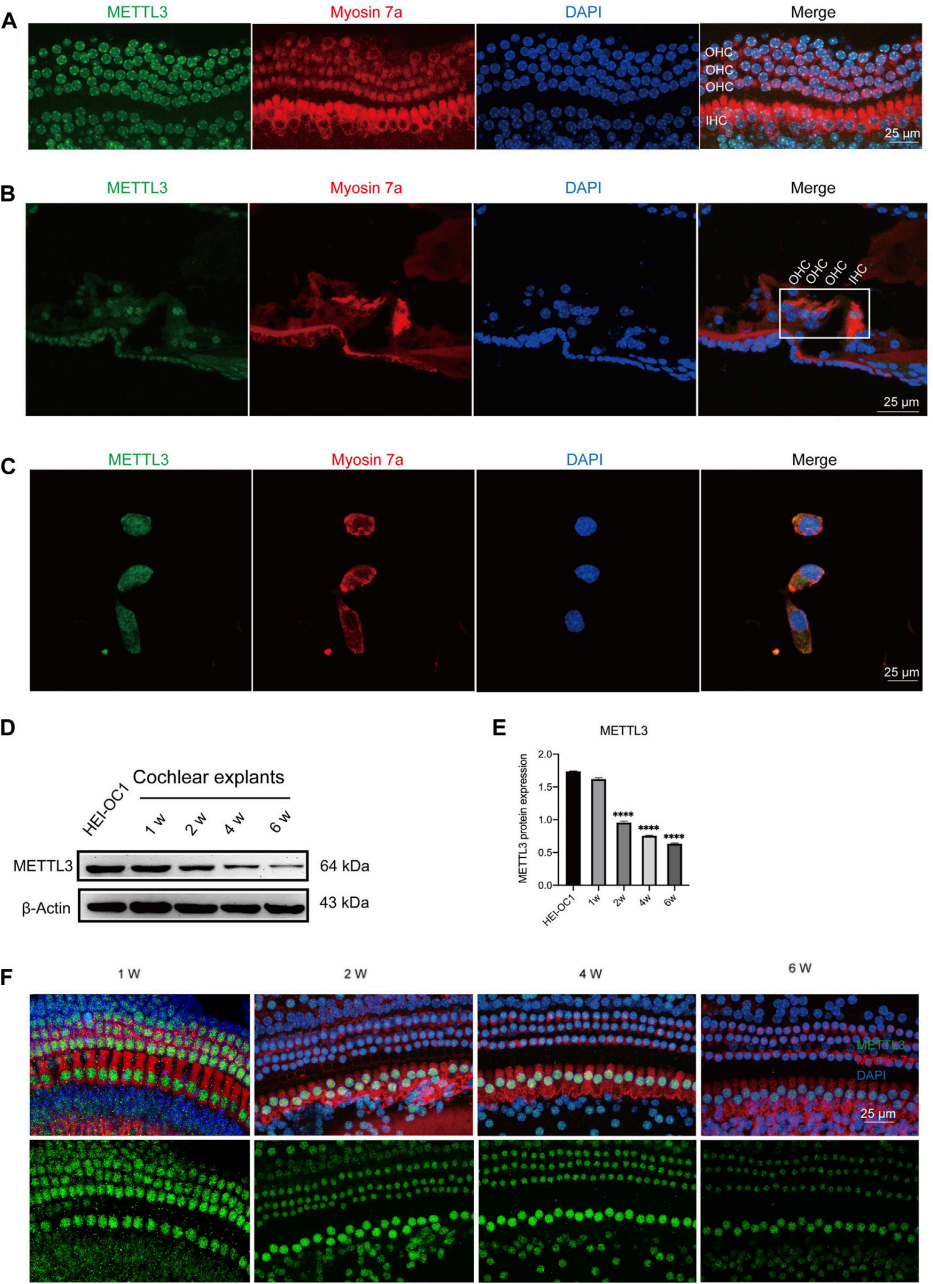
Expression of METTL3 assessed in neonatal cochlear explants and HEI-OC1 cells. **(A)** Immunofluorescence staining revealed robust expression of METTL3 (green) in the hair cells of cochlear explants, including outer hair cells (OHCs) and inner hair cells (IHCs). **(B)** Immunofluorescence analysis of frozen sections of the organ of Corti highlighted the presence of METTL3 (green) in the four rows of hair cells. **(C)** METTL3 expression was also prominently observed in HEI-OC1 cells via immunofluorescence. **(D)** Western blotting confirmed the expression of METTL3 in both cochlear explants and HEI-OC1 cells. **(E)** Quantitative analysis revealed a decrease in METTL3 expression in cochlear explants with age, as indicated by a significant reduction observed in the 1-week group compared to other groups, with comparisons made by quantifying the gray values between different groups (n = 4 for each group; ****P < 0.0001 vs. the 1w group; ANOVA). **(F)** Immunofluorescence images depicted the expression pattern of METTL3 in cochleae of different ages.

Expression levels of METTL3 were identified in the nucleus of cells through immunostaining of neonatal cochlear explants (Figure 3A), murine cochlea sections (Figure 3B), and HEI-OC1 cells (Figure 3C). Western blotting analysis confirmed the expression of METTL3 in neonatal cochlear explants and HEI-OC1 cells (Figures 3D, E). A decline in METTL3 expression was observed with increasing age, from postnatal day 7 (P7) to P42, in cochlear explants, particularly in outer hair cells (Figure 3F). These findings substantiate the expression of METTL3 in cochlear explants and HEI-OC1 cells, providing a foundational basis for subsequent research.

### 3.3 Decrease in METTL3 expression in hair cells and HEI-OC1 cells following kanamycin treatment

**FIGURE 4:**
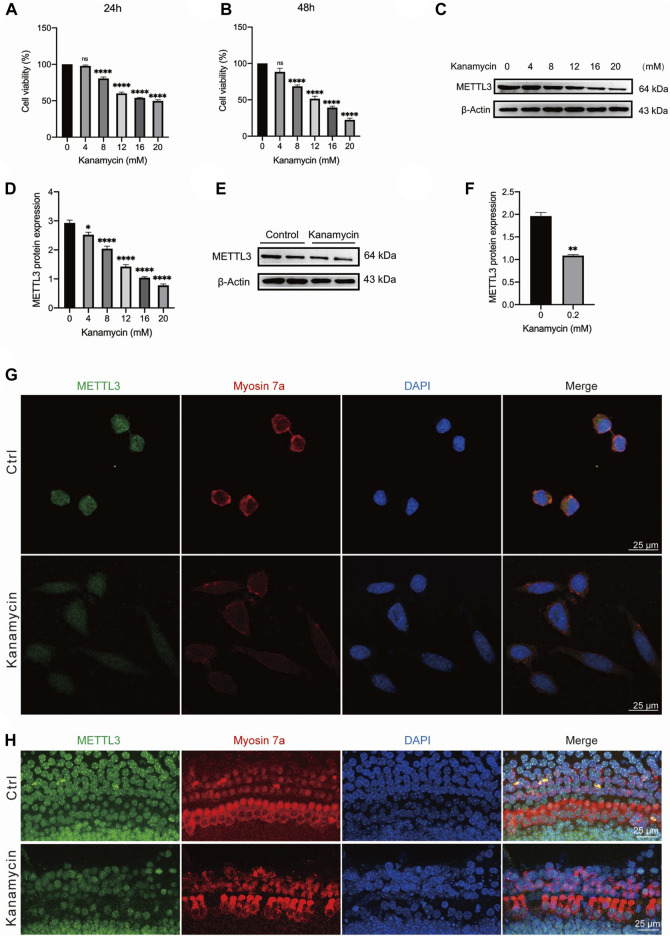
Kanamycin treatment induces a reduction in the expression level of METTL3 in both HEI-OC1 cells and cochlear hair cells. The control groups comprised of neonatal cochlear explants or HEI-OC1 cells without kanamycin treatment, while those subjected to kanamycin treatment constituted the kanamycin groups. **(A, B)** Cell viability of HEI-OC1 cells was assessed under various concentrations of kanamycin treatment for 24 and 48 h using CCK-8 kits. Statistical analysis revealed a significant decrease in cell viability in the kanamycin-treated groups compared to those without kanamycin treatment (n = 4 for each group; ****P < 0.0001; vs. the group without kanamycin treatment; ANOVA). **(C, D)** Western blotting analysis and semi-quantitative assessment depicted a decrease in METTL3 protein expression in HEI-OC1 cells treated with different concentrations of kanamycin for 24 h. The reduction in METTL3 expression was statistically significant compared to the group without kanamycin treatment, assessed by comparing their gray values (n = 4 for each group; *P < 0.05; ****P < 0.0001; vs. the group without kanamycin treatment; ANOVA). **(E, F)** Similarly, Western blot analysis revealed a decrease in METTL3 expression in neonatal cochlear explants treated with 0.2 mM kanamycin for 24 h compared to those treated with normal culture medium, as indicated by the difference in gray values between them (n = 4 for each group; **P < 0.01; vs. the control group without kanamycin treatment; unpaired, two-tailed t-test). **(G)** Immunofluorescence staining demonstrated a reduction in METTL3 expression in kanamycin-treated HEI-OC1 cells. **(H)** Immunofluorescence staining of cochlear explants indicated a decrease in METTL3 expression following kanamycin treatment.

HEI-OC1 cells were exposed to varying concentrations of kanamycin (0, 4, 8, 12, 16, or 20 mM) for 24 and 48 h. Cell viability decreased progressively with higher doses and longer exposure durations. Optimal impairment of HEI-OC1 cells was observed with 20 mM kanamycin for 24 h, resulting in a significant reduction in cell viability to 49.8% ± 1.3% (n = 4) (Figures 4A, B). Western blotting analysis conducted during the concentration gradient tests revealed a reduction in METTL3 levels with increasing kanamycin concentration (Figures 4C, D).

For *in vitro* experiments, cochlear explants isolated from P3 C57BL/6 mice were utilized. Western blotting results demonstrated a decrease in the expression level of METTL3 in neonatal cochlear explants treated with 0.2 mM kanamycin for 24 h (Figures 4E,F; n = 4). Additionally, immunostaining revealed a reduction in METTL3 expression in kanamycin-treated HEI-OC1 cells following treatment with 20 mM kanamycin (Figure 4G). Furthermore, compared to untreated cells, the morphology and arrangement of cochlear hair cells were disrupted post-kanamycin treatment (Figure 4H).

### 3.4 METTL3 expression increased kanamycin-induced SG formation in HEI-OC1 cells

**FIGURE 5:**
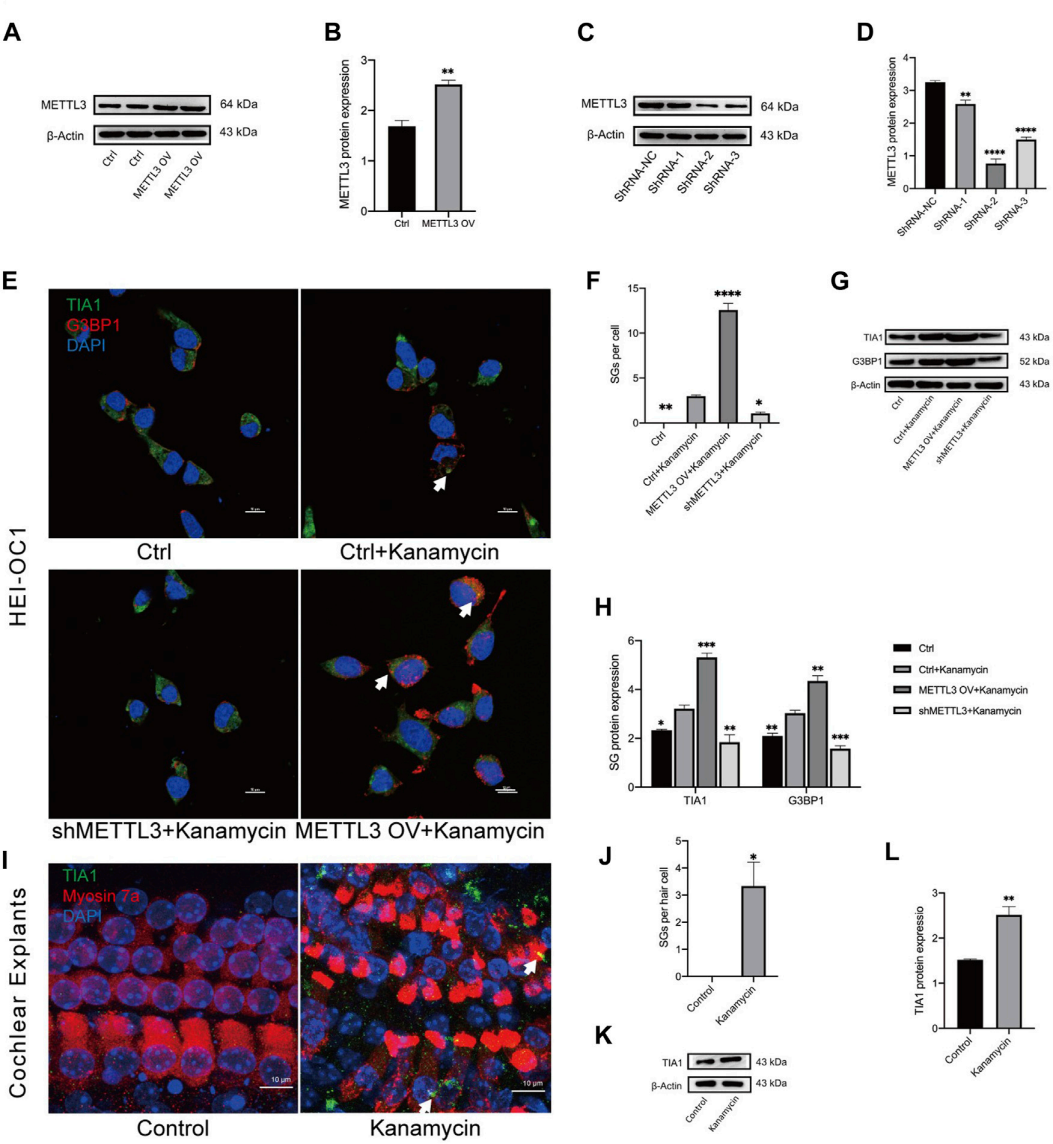
Kanamycin treatment induces SG formation in both HEI-OC1 cells and cochlear hair cells. **(A–D)** Western blotting analysis was used to assess the expression levels of METTL3 in HEI-OC1 cells following lentiviral transfection. Cells transfected with the empty lentivirus vector served as the control group, while those overexpressing METTL3 were designated as the METTL3 OV group. Cells transfected with negative shRNA were labeled as the ShRNA-NC group, with three different sequences of METTL3 shRNA defined as ShRNA-1, ShRNA-2, and ShRNA-1, respectively. Statistical analysis revealed significant differences compared to the ctrl or shRNA-NC group (n = 4 for each group; **P < 0.01; ****P < 0.0001). **(E, F)** SGs were visualized through double-immunostaining of anti-TIA1 (green) and anti-G3BP1 (red) in different groups and quantified accordingly. HEI-OC1 cells untreated with kanamycin comprised the ctrl group, whereas those exposed to kanamycin were termed the ctrl + kanamycin group. METTL3 OV represented the METTL3 overexpression HEI-OC1 cell line, while shMETTL3 denoted the METTL3 knockdown HEI-OC1 cell line. Statistical analysis revealed significant differences compared to the ctrl + kanamycin group (n = 4 for each group; *P < 0.05; **P < 0.01; ****P < 0.0001). **(G, H)** Protein expression levels of TIA1 and G3BP1 were assessed via Western blotting in the Ctrl, Ctrl + kanamycin, shMETTL3 + kanamycin, and METTL3 OV + kanamycin groups. Statistical analysis revealed significant differences compared to the ctrl + kanamycin group (n = 4 for each group; *P < 0.05; **P < 0.01; ***P < 0.001; ****P < 0.0001). **(I, J)** TIA1 (green) was co-stained with DAPI (blue) and anti-myosin 7a (red) in neonatal cochlear explants, and SGs were quantified accordingly. Neonatal cochlear explants untreated with kanamycin constituted the control group, while those treated with kanamycin comprised the kanamycin group. Statistical analysis revealed significant differences compared to the control group (n = 4 for each group; *P < 0.05). **(K, L)** Western blotting analysis demonstrated increased TIA1 expression in cochlear explants following kanamycin treatment. Statistical analysis revealed significant differences compared to the control group (n = 4 for each group; *P < 0.05; **P < 0.01). The unpaired, two-tailed t-test was applied in Figures 5B, J, L, while ANOVA was used in Figures 5D, F, H. Quantification of Western blot results involved comparing the differences in gray values or fluorescence intensities as referred to in Figures 5B, D, F, H, J, L.

To investigate the impact of METTL3 on kanamycin-induced ototoxicity, METTL3 was modulated through lentiviral transfection in HEI-OC1 cells. The control (Ctrl) group consisted of untreated cells, while cells transfected with nonsense shRNA served as the shRNA-NC group. Post-transfection, METTL3 protein levels were confirmed via Western blotting analysis (Figures 5A–D; METTL3 OV denotes the METTL3 overexpression HEI-OC1 cell line, while shMETTL3 represents the METTL3 knockdown HEI-OC1 cell line).

To assess the influence of METTL3 expression on SG formation, TIA1 and G3BP1 were co-stained in all groups. Kanamycin-induced punctate expression of TIA1 and G3BP1 was observed in the cell cytoplasm (Figure 5E). Notably, the METTL3 OV + kanamycin group exhibited the highest number of SGs among all four groups (Figure 5F). Furthermore, Western blotting analysis revealed a decrease in the expression of TIA1 and G3BP1 in the shMETTL3 + kanamycin group, whereas an increase was observed in the METTL3 OV + kanamycin group following kanamycin treatment (Figures 5G, H). In neonatal cochlear explants, TIA1 was co-stained with myosin 7a and DAPI. Kanamycin treatment induced the formation of TIA1-positive SGs in cochlear hair cells (Figures 5I, J). These results were further corroborated by Western blotting analysis conducted on cochlear explants (Figures 5K, L).

### 3.5 METTL3 inhibited kanamycin-induced ROS and apoptosis

**FIGURE 6:**
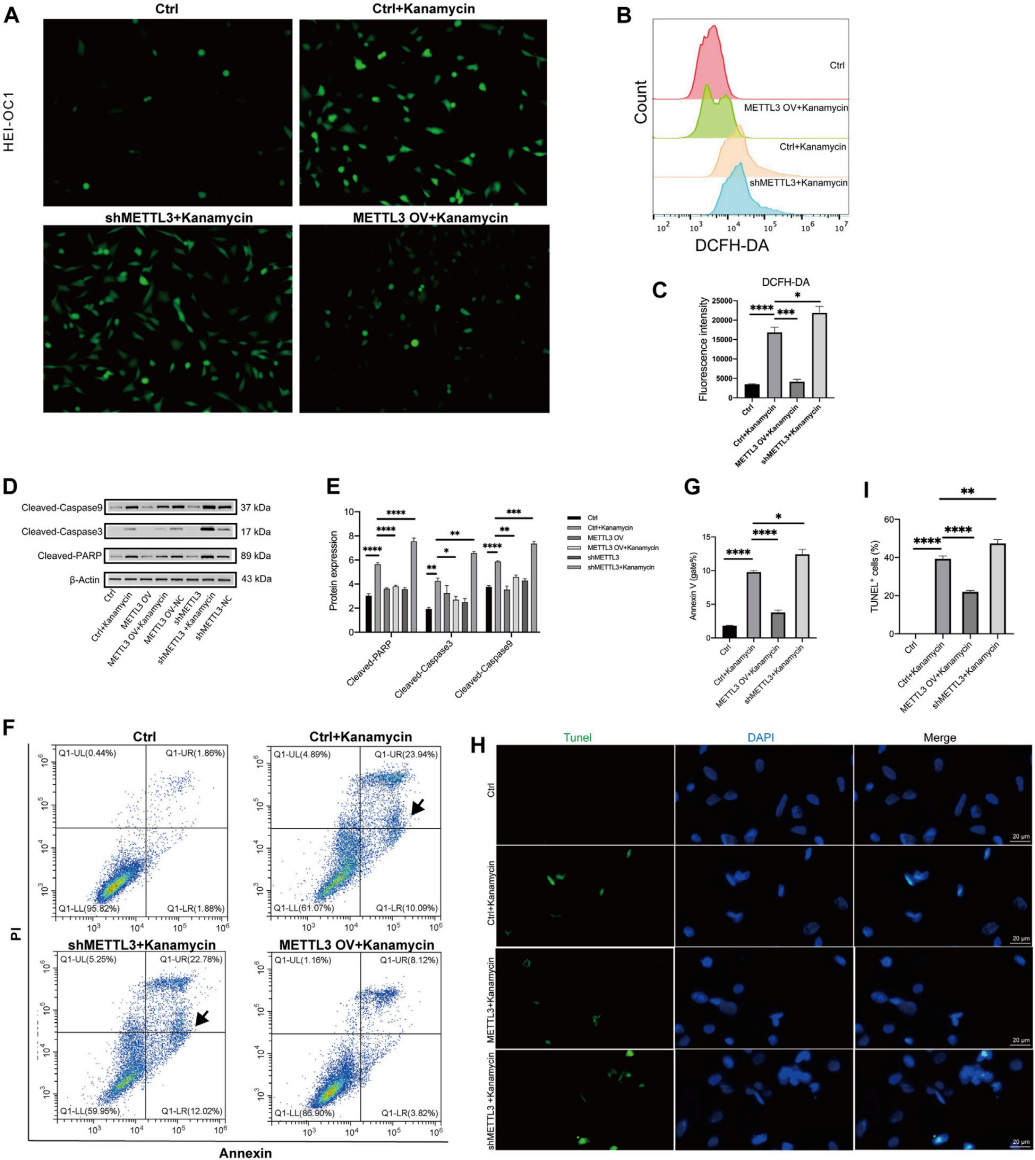
METTL3 expression attenuates apoptosis and ROS generation in kanamycin-treated HEI-OC1 cells. The ctrl group comprised of HEI-OC1 cells without kanamycin treatment, while those treated with kanamycin constituted the ctrl + kanamycin group. METTL3 OV represented the METTL3 overexpression HEI-OC1 cell line, whereas shMETTL3 indicated the METTL3 knockdown HEI-OC1 cell line. **(A)** Intracellular ROS accumulation in HEI-OC1 cells was assessed using a DCFH-DA staining kit. **(B, C)** ROS levels in the Ctrl, Ctrl + kanamycin, shMETTL3 + kanamycin, and METTL3 OV + kanamycin groups were determined through DCFH-DA fluorescent probe staining. Statistical analysis revealed significant differences compared to the ctrl group (n = 4 for each group; *P < 0.05; **P < 0.01). ANOVA was used for analysis. **(D, E)** Protein expression levels of cleaved caspase-3, cleaved caspase-9, and cleaved PARP were assessed under different conditions using Western blotting and semi-quantification analyses. Statistical analysis revealed significant differences compared to the ctrl + kanamycin group (n = 4 for each group; *P < 0.05; **P < 0.01; ***P < 0.001; ****P < 0.0001). ANOVA was used for analysis. **(F, G)** Proportions of apoptotic cells in the different groups were assessed via flow cytometry analysis. Statistical analysis revealed significant differences compared to the ctrl + kanamycin group (n = 4 for each group; *P < 0.05; ****P < 0.0001). ANOVA was used for analysis. **(H, I)** Apoptosis levels were determined using TUNEL assay. Statistical analysis revealed significant differences in fluorescence intensity compared to the control + kanamycin group. (n = 4 for each group; ****P < 0.0001). ANOVA was used for analysis.

A DCFH-DA staining kit was used to assess ROS production levels, revealing an increase in ROS following treatment with 20 mM kanamycin for 24 h (Figure 6A). To investigate the association between kanamycin-induced ototoxicity and ROS, the proportion of DCFH-DA was assessed in HEI-OC1 cells using a fluorescent probe (Figures 6B, C). Flow cytometry analysis demonstrated that the shMETTL3 + kanamycin group exhibited the highest fluorescence intensity of DCFH-DA compared to other groups, the greatest accumulation of ROS among all groups.

Western blotting analysis revealed significant decreases in the expression levels of cleaved caspase-3, cleaved caspase-9, and cleaved PARP in the METTLE3 OV + kanamycin group, while significant increases were observed in the shMETTLE3 + kanamycin group (Figures 6D, E). The number of apoptotic cells in each group was assessed using flow cytometry. Annexin V-positive and PI-negative cells in the lower right quadrant of the plots were defined as early apoptotic cells and were used in the statistical analysis. Compared to the Ctrl + kanamycin group, the METTLE3 OV + kanamycin group exhibited significantly lower levels of apoptosis, while the shMETTLE3 + kanamycin group displayed significantly higher levels of apoptosis (Figures 6F, G). Furthermore, the number of TUNEL-positive cells increased in the kanamycin + shMETTL3 group, whereas it decreased in the METTLE3 OV + kanamycin group (Figures 6H, I). These findings indicate that METTL3 knockdown enhances apoptosis and ROS generation induced by kanamycin treatment, while METTL3 overexpression inhibits kanamycin-induced apoptosis and ROS generation in HEI-OC1 cells.

## 4 Discussion

Aminoglycoside antibiotics are essential therapeutics for managing challenging infectious diseases; however, their inherent risk of ototoxicity poses a substantial challenge. Our findings indicate that genetic modulation of METTL3 expression may mitigate aminoglycoside-induced ototoxicity, potentially mediated through the enhancement of SGs.

Among the myriad mRNA modifications observed in eukaryotes, m6A is one of the most prevalent (Wiener and Schwartz, 2021). The objective of our research was to elucidate the involvement of METTL3 in safeguarding against ototoxicity. By examining METTL3 expression levels in cochlear explants and HEI-OC1 cells, we observed a reduction in METTL3 expression following kanamycin-induced damage. The correlation between kanamycin treatment and METTL3 expression suggests the existence of an intermediary mechanism, laying the groundwork for subsequent assessment of the roles of METTL3 in kanamycin-induced ototoxicity.

In response to hypoxia, heat stimulation, and various other stressors, eukaryotic cells form SGs within the cytoplasm. Composed primarily of mRNA and mRNA-binding proteins, SGs sequester mRNA molecules that stall during the initial stages of translation, thereby modulating RNA translation processes (Youn et al., 2019). SG formation plays a pivotal role in regulating stress responses, combating viral infections, and dampening apoptosis-related signaling pathways. This mechanism reduces cellular damage induced by stressors, promoting cellular adaptation and survival under adverse conditions (Mateju and Chao, 2022). Professor Kang identified that G3BP exhibits antioxidant properties, and SGs function to inhibit apoptosis by reducing ROS production (Kang et al., 2021). Conversely, disruption of SG formation during stress can increase cell mortality. SGs have also been proven to confer protective effects in numerous diseases, including early acute ischemic stroke (Si et al., 2020). Previous studies have demonstrated that treatment with aminoglycosides induces SG formation in mammalian cochlear hair cells. Notably, drug-induced SGs have been found to enhance the survival of hair cells (Gonçalves et al., 2019b; Gonçalves et al., 2019a).

The involvement of m6A methylation in SG formation has been firmly established under various conditions (Vaid et al., 2023). m6A modification is considered enriched within SGs, and deletion of YTHDF1/3 has been shown to impede SG formation and mRNA recruitment (Fu and Zhuang, 2020a). Therefore, the YTHDF protein family emerges as pivotal regulators of SG formation. (Fu and Zhuang, 2020a). In prostate cancer, silencing androgen receptor mRNA delays the formation of SGs induced by androgen receptor pathway inhibition (ARPI) stress (Somasekharan et al., 2022). m6A methylation interferes with RNA binding by SG-associated proteins like G3BP1/2, YTHDF1, CAPRIN1, and RBM42 (Fu and Zhuang, 2020b). However, the specific role of m6A or METTL3 in the mechanism underlying stress granule-mediated protection and kanamycin-induced ototoxicity in cochlear hair cells remains unclear.

We observed the formation of SGs and a reduction in METTL3 expression in mice subjected to kanamycin treatment. In HEI-OC1 cells, kanamycin induced apoptosis mediated by ROS, while METTL3 expression augmented SG formation and attenuated the apoptotic rate. Based on these observations, our objective was to ascertain the involvement of METTL3 expression and SGs in ROS-mediated apoptosis induced by kanamycin. To contextualize the *in vivo* findings, we established ototoxic models induced by kanamycin in both HEI-OC1 cells and cochlear explants. A concentration of 0.2 mM kanamycin was administered to neonatal cochlear explants based on dose-response assessments (ranging from 0.2 to 1 mM), as visible hair cells were eliminated following a 24-hour treatment with 0.2 mM kanamycin (Liu et al., 2015). Similarly, a concentration of 20 mM kanamycin, which reduced cell viability to 50%, was applied to HEI-OC1 cells based on dose-response assessments (ranging from 0 to 20 mM) using the CCK8 assay.

*In vitro* experiments corroborated our *in vivo* findings, demonstrating a decrease in METTL3 expression and induction of SG formation following kanamycin treatment. Moreover, METTL3 overexpression facilitated SG formation, whereas downregulation of METTL3 mitigated SG formation. Additionally, mounting evidence implicates ROS-induced apoptosis in the pathogenesis of aminoglycoside-induced ototoxicity (Kim et al., 2021; Ogier et al., 2022). Accordingly, the expression levels of cleaved caspase-3, cleaved caspase-9, cleaved PARP, and ROS increased upon METTL3 knockdown, while they decreased upon METTL3 overexpression in HEI-OC1 cells treated with kanamycin. These findings indicate a pivotal role for METTL3 in inhibiting ROS-mediated ototoxicity by promoting SG formation, underscoring the therapeutic potential of targeting METTL3 in mitigating aminoglycoside-induced ototoxicity.

Prior studies have established that METTL3 expression augments SG formation, which serves as a protective mechanism against cellular stress (Si et al., 2020). Concurrently, ROS and apoptosis constitute fundamental mechanisms underlying drug-induced ototoxicity. Our study revealed that METTL3 expression promotes SG formation while decreasing ROS levels and apoptosis, thereby mitigating ototoxic damage. Collectively, these findings imply the existence of a METTL3/SG/ROS/apoptosis axis implicated in hair cell ototoxicity, indicating a new therapeutic target for protective interventions.

However, detailed mechanistic studies at the molecular level to elucidate how METTL3 mediates SG formation and its role in reducing ROS and apoptosis were not examined in this study, representing a primary limitation. Further research is warranted to delineate the precise role of METTL3 in the pathogenesis of deafness. Additionally, this study exclusively used kanamycin-induced auditory damage in hair cells. Future investigations should consider whether METTL3 has protective effects against other ototoxic drugs and conditions.

## 5 Conclusion

This study underscores the critical role of METTL3 in mitigating kanamycin-induced ototoxicity by inhibiting ROS and apoptosis through the promotion of SG formation. These findings offer new insights into potential therapeutic strategies for mitigating aminoglycoside-induced ototoxicity, highlighting the therapeutic potential of targeting epigenetic mechanisms and SGs.

## Data Availability

The raw data supporting the conclusions of this article will be made available by the authors, without undue reservation.
